# Feasibility Study of Digital Image Correlation in Determining Strains in Concrete Exposed to Fire

**DOI:** 10.3390/ma13112516

**Published:** 2020-05-31

**Authors:** Katarzyna Mróz, Marcin Tekieli, Izabela Hager

**Affiliations:** 1 Chair of Building Materials Engineering, Faculty of Civil Engineering, Cracow University of Technology, 31-155 Cracow, Poland; izabela.hager@pk.edu.pl; 2 Chair for Computational Engineering, Faculty of Civil Engineering, Cracow University of Technology, 31-155 Cracow, Poland; mtekieli@pk.edu.pl

**Keywords:** concrete, fire, concrete spalling, strain, digital image correlation

## Abstract

Concrete is prone to spalling when exposed to fire. During fire tests, the strains of concrete elements are hard to identify, both from the fire-exposed face and the non-exposed face. This paper presents a field experiment which employed the original CivEng digital image correlation (DIC) method developed at the Cracow University of Technology to measure the strain fields of elements exposed to heating by a pin-point gas burner. The paper presents experimental proof that it is possible to analyze the deformation of both unheated and fire-exposed sides of heated concrete by the DIC method. The strain fields, crack patterns and modes of crack development are presented. The study shows the encouraging results of employing the DIC method to test concrete behavior under fire attack.

## 1. Introduction

The thermal instability of concrete is one of the most important phenomena that take place during fire exposure on concrete structures. Concrete disintegrates explosively or gradually in the first 15 min of fire in the temperature range between 150 and 300 °C. To describe this phenomenon, the fire spalling term is used frequently by the civil engineering community. Experimental tests on concrete’s sensitivity to fire spalling are becoming more complex and still delivering new data for analytical and numerical models [1,2,3]. One of the most interesting issues relates to the prediction and modeling of the deformation of concrete during fire exposure. The most common experimental tests of concrete’s susceptibility to fire spalling consist of heating a concrete element (e.g., a rectangular or circular slab) from one side with the standard (ISO 834-1 [4]) or hydrocarbon (Eurocode 1 [5]) fire scenario. The element is placed in front of the furnace opening and insulated with ceramic wool at its perimeter. The fire, developed in the furnace, acts on the concrete element through the opening. The tested concrete element may be unloaded, loaded bilaterally or unilaterally in compression, or restrained to its thermal expansion with a steel rim [1]. During such experimental tests, the concrete is investigated to understand its material behavior during fire exposure. If spalling occurs during the time of fire exposure, the start of the spalling event, its duration, and its intensity are described [2,6]. After the fire test, the maximum spalling depth, its volume, or its average depth is presented [2,7]. Additionally, the thermocouples are placed at different depths in the concrete section to measure the temperature development inside the concrete element. Sometimes, the tests are enriched by further measurements; for example, embedded gauges placed at different distances to measure the pore pressure changes inside the concrete or linear voltage displacement transducers (LVDTs) installed at the exterior side of the element to monitor the deformation of the concrete element (especially the deflection) [8].

With regards to the measures taken during and after the fire test, the accurate and reliable monitoring of the deformation of the concrete element due to fire exposure is of high importance, especially for further use in numerical models. The methods of monitoring concrete deformation at an elevated temperature can be divided into those requiring direct contact with the tested element and those that monitor deformation without contact. Le et al. [9] presented a brief review of contact and non-contact methods. Thus far, in the case of experimental tests on the fire spalling of concrete, contact methods have been more frequently used.

In terms of the contact methods that are more frequently used while testing concrete under fire action, we can distinguish between them as follows:

- Linear voltage displacement transducers (LVDTs): LVDTs are used to measure the vertical deformation of the unheated part of the concrete. They are mounted on the opposite side of the heated surface, in several places and in two directions. They are commonly used to monitor displacement toward and away from the source of fire (i.e., the sagging and hogging effects). Miah [3] investigated the effect of compressive loading on the fire spalling of concrete. An LVDT was successfully used to measure the deflection of the concrete slabs during the tests. A major limitation of this method is that the measurements can only be taken at discrete points.
- Strain gauges/high-temperature strain gauges: strain gauges are commonly used to measure concrete deformation on a horizontal plane (displacement at the concrete surface), in structural and material testing. In the case of the testing of concrete under fire exposure, the bond between concrete and gauge is weakened by evaporating water, flames, and an elevated temperature, even on the unexposed side. Standard gauges may work up to ca. 100 °C, while high-temperature strain gauges are described as withstanding ca. 1000 °C, but the severity and change of the condition mean that the concrete–gauge bond cannot be considered reliable. The measuring grid is highly prone to any change in temperature or moisture state [10,11].

Non-contact measurement methods are used to expand the recording capabilities using inductive sensors or strain gauges. By using them, it is possible to register the full-field displacement of the surface of the tested specimen while maintaining a high resolution and the stability of the measurement.

One of the most widely used non-contact measuring techniques is the digital image correlation (DIC) method. Nowadays, the DIC method is widely used to determine the full deformation fields of various materials or composites, which can be used to better characterize and describe the mechanisms of the deformation of these materials. The DIC method is based on the mutual correlation of digital images of the specimen surface taken during the test. From the images taken during the test, the region of interest (ROI) is separated from the image background. When generating full-displacement or deformation fields, the ROI is divided into smaller grid elements. Elements of such a grid are referred to as subsets or markers in the shape of squares or rectangles. Visualization of the displacements of grid elements can be conducted using the vector field or displacement paths assigned to each grid element. Based on the obtained displacements of the grid elements, in the first place, a full displacement field can be generated, based on which a full deformation field can be determined. Nevertheless, digital methods present strict requirements to ensure proper data acquisition. Particularly, they require the researcher to ensure enough disk volume for data storage or to avoid environmental instability; for example, moving shadows, excessive sunlight, or wind.

In the case of testing concrete behavior under fire exposure, the DIC method was only previously used for the small-scale preliminary studies conducted by Le et al. [9]. In the experiment presented in [9], the vertical deformation of the loading cylinder was monitored using the DIC method. Then, the results were compared to the displacement of the loading actuator. The researchers achieved a good compatibility between DIC measurement and displacement of the loading actuator.

**Figure 1:**
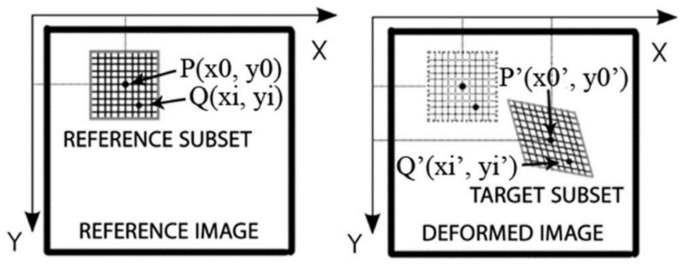
Image subsets before and after deformation [14].

The optical measurement system (CivEng Vision) used in this research was previously developed by Tekieli and Słoński at Cracow University of Technology [12,13]. In the laboratory of the Cracow University of Technology, the DIC method is widely used for tests for many purposes. Both brittle and flexible materials can be investigated [14]. The pictures taken are used as a 2D matrix of pixels and each picture is correlated with the reference one (Fig. 1). The points of the grid based on the specified image subsets are matched and identified as that associated with the highest value of the correlation coefficient. This coefficient is calculated between the reference subset “f” and the target subset “g”, whose dimensions are equal and are M × N pixels using the zero-mean normalized cross-correlation method, as described by Equation (1), where *u_f_* is the intensity of the reference subset and *u_g_* is the intensity of the target subset form (Figure 1). A detailed description of the method is presented in [14].

(1) $${\mathrm{CC}}^{\mathrm{ZMN}}=\frac{\sum_{i=1}^{M}\sum_{j=1}^{N}(\left(f\left(i,j\right){-u}_{f}\right)\times (g\left(i,\;j\right){-u}_{g}))}{\sqrt{\sum_{i=1}^{M}\sum_{j=1}^{N}{(f\left(i,j\right){-u}_{f})}^{2}}\times \sqrt{\sum_{i=1}^{M}\sum_{j=1}^{N}{(g\left(i,j\right){-u}_{g})}^{2}}}$$

Within the framework of the presented research, tests on the fire spalling of concrete were carried out. To present the usefulness and feasibility of the DIC measurements while testing concrete’s susceptibility to fire spalling, concrete specimens of two different sizes were employed. The flame from a propane-butane torch acted on the concrete element from one side while the DIC measurement was taken from both the fire-exposed and unexposed surfaces. The presented results show that DIC is a reliable method that can be used in such a number of the measurement points that may represent the displacement field for the entire surface. Moreover, the development of cracks during the fire test may be tracked.

## 2. Materials and Methods

### 2.1. Materials

In this study, high strength concrete (HSC) made with basalt aggregate was tested. The detailed composition is given in Table 1. The concrete was made with ground granulated blast-furnace slag cement CEM III and characterized by the water to cement (w/c) ratio of 0.3.

The small testing specimens that were 0.3 m × 0.3 m × 0.2 m in size were manufactured and cut into two sizes: 0.2 cm × 0.2 cm × 0.1 m and 0.3 m × 0.3 m × 0.1 m. The specimens were cut 30 days before carrying out the fire exposure tests. The specimens were placed in plastic molds for 24 h and for the next 7 days were protected from drying with a plastic cover. Subsequently, the specimens were stored until the test in air-drying conditions.

The material properties of the manufactured concrete were determined after 90 days of curing in air-drying conditions. The physical properties (density, water content, and gas permeability (Cembureau method)) and the mechanical properties (compressive strength and modulus of elasticity) were tested using standard concrete specimens. The values obtained for the initial properties are presented in Table 2.

### 2.2. Fire Exposure

**Figure 2:**
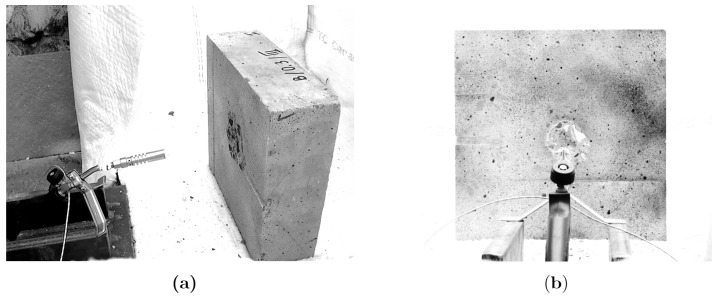
The experimental test stand and the concrete element with a size of 0.3 m × 0.3 m × 0.1 m: (**a**) Side view; (**b**) Front view.

**Figure 3:**
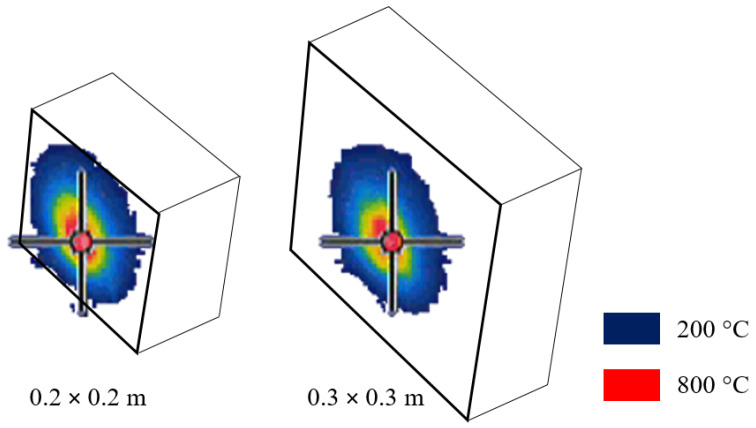
The directly fire-affected zones for the tested concrete element.

The experimental test method was inspired by the reaction to fire tests—part 2: single-flame source test, according to ISO 11925-2b [15]. The single test consisted of using a small propane-butane gas burner (torch) placed in front of the concrete element (Figure 2). In all tests, the angle and the distance between the burner and the specimens were the same. The mean flame temperature at the concrete surface was measured with a thermal camera as ca. 800 °C and remained constant during the test. Additionally, the thermal camera was used to point out the area that was directly exposed to the fire. Figure 3 presents the directly affected circle zones with respect to the size of the specimen. The zones with a temperature higher than 200 °C as observed with the infrared camera are marked in dark blue, while the temperature of 800 °C is marked in red. The directly fire-exposed area was a round surface with diameter ca. Ø0.15 m regardless of the element size. The single test lasted 20 min.

### 2.3. Digital Image Correlation Method

**Figure 4:**
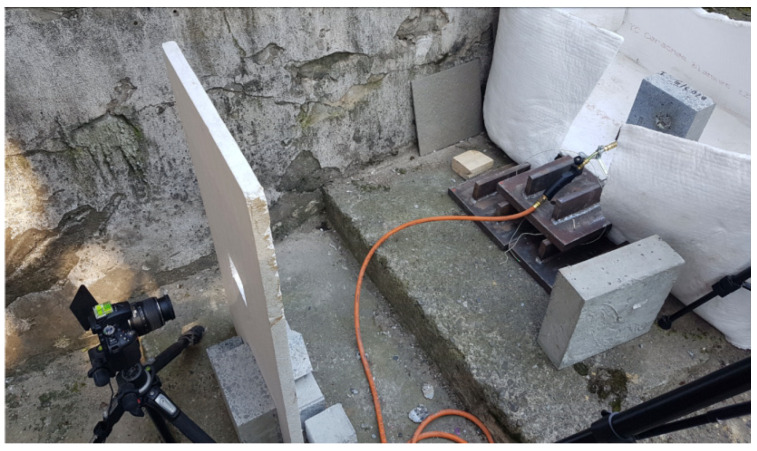
Test stand used for the simple flame test.

**Figure 5:**
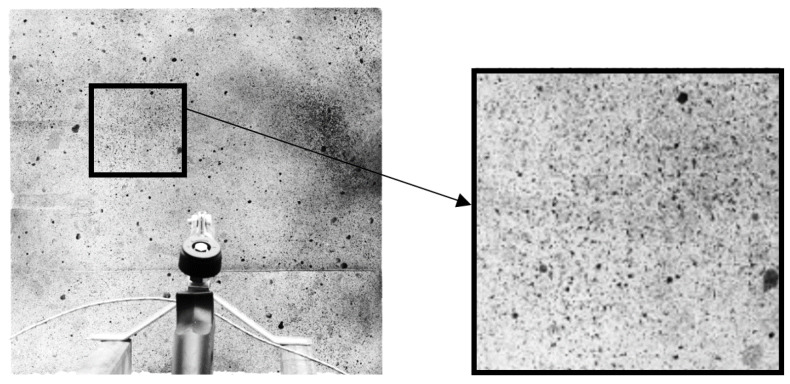
Pattern of randomly distributed black dots to be tracked by digital image correlation (DIC).

In the case of a fire test with the use of a small torch, it is possible to take pictures in front of the fire-exposed side. The test stand employed is presented in Figure 4. Due to the dynamic destruction of the samples, the camera on the heated side was protected with a styrofoam shield cover with a hole for the camera lens. The surface of the specimen was prepared in a similar way to the standard approach, but heat-resistant black spray paints were used to produce a random black and white pattern as shown in Figure 5.

To track the deformation of the tested specimen, the DIC method was used. In this case, the DIC method relied on the correlation of the subsequent digital pictures taken while exposing the concrete to fire from two surfaces: the one that was directly exposed to fire and the one on the opposite side to the flames.

**Figure 6:**
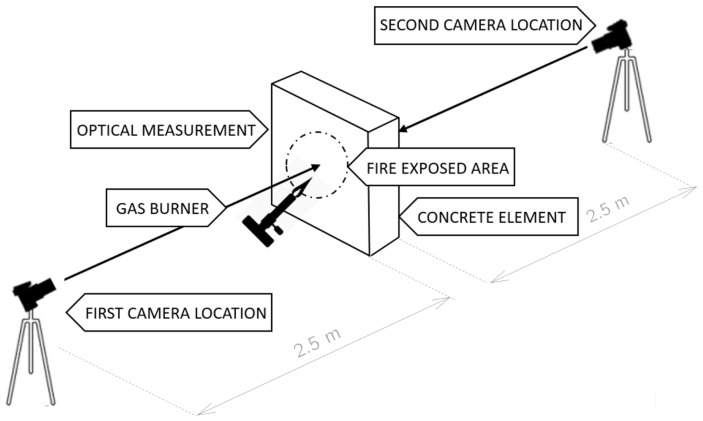
Scheme of the experimental stand.

Each specimen was observed using two DSLR cameras connected by one automatic trigger: one set on the heated side and one located on the opposite side (Figure 6). The pictures were taken every 10 s during fire exposure.

After the test, the grid of subsets was defined. Due to the spalling events, the front side of the specimen could only be analyzed in the area unaffected by spalling, while the entirety of the opposite side could be analyzed.

Sub-pixel interpolation was employed to improve the resolution of the optical measurement. The sub-values were calculated by cubic spline interpolation, which enabled the measurement resolution level to be increased to 1/100 pixel. The displacements of the subset were computed by converting px to mm. Before the test, the transition factor was determined with the use of the calibration form.

## 3. Results

### 3.1. Deformation of the Heated Side

**Figure 7:**
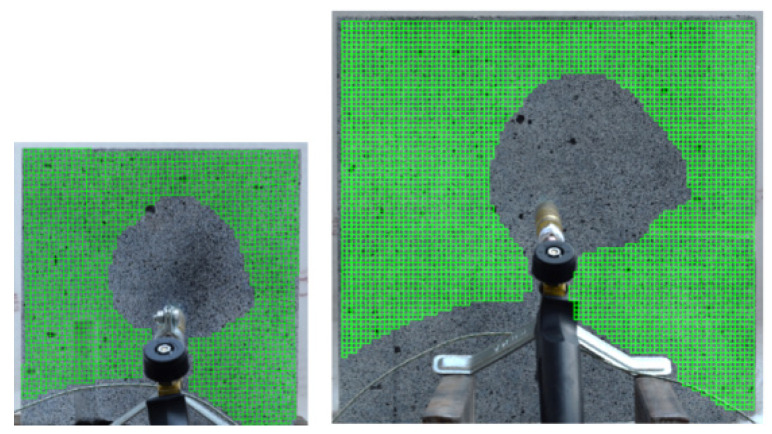
Grid of subsets in medium (200 × 200 mm) and large (300 × 300 mm) specimens.

Since the central part of the specimen was very quickly destroyed by the falling pieces of concrete and the random pattern was degraded, the masking procedure was used on the deformation maps so that only the effects around the damaged fragment were visible (Figure 7).

**Figure 8:**
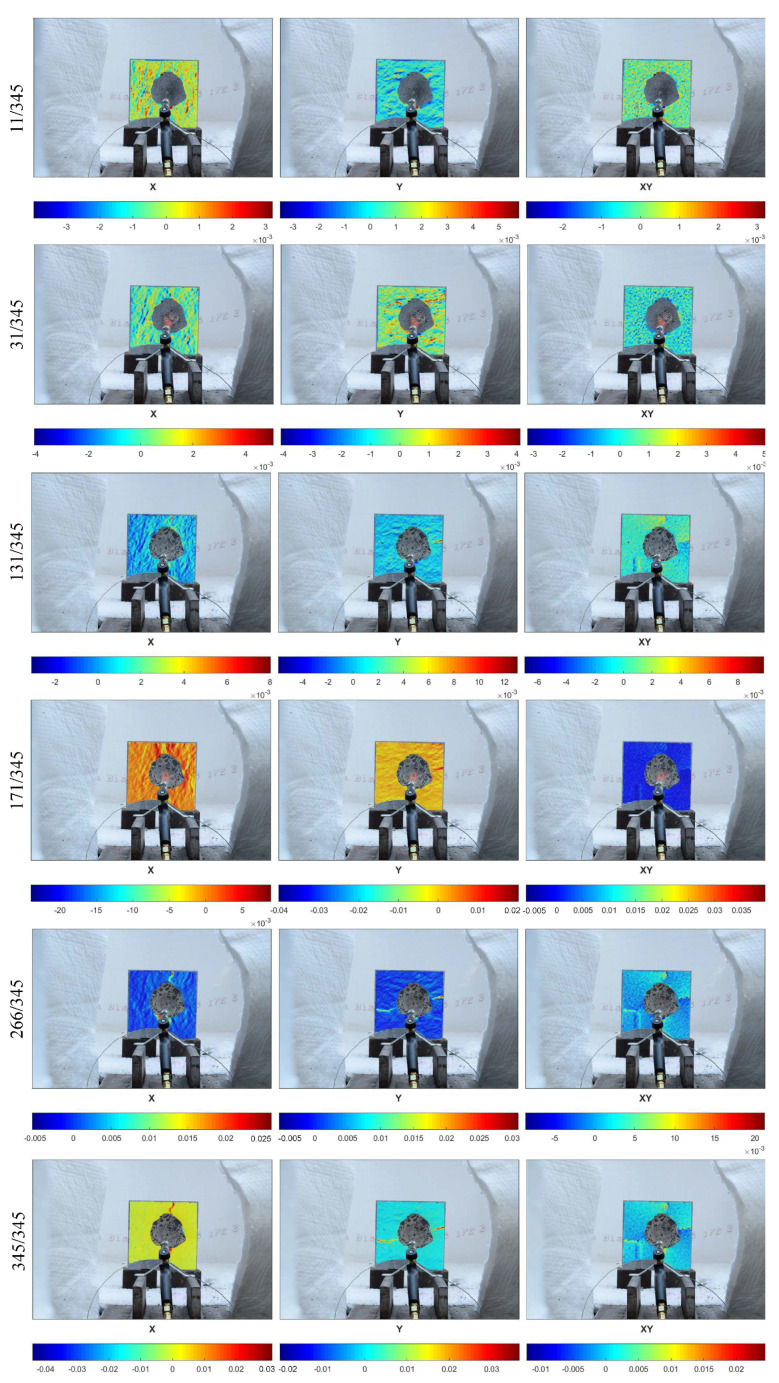
Deformation fields in the x- and y-directions for the medium specimen (200 × 200 mm).

**Figure 9:**
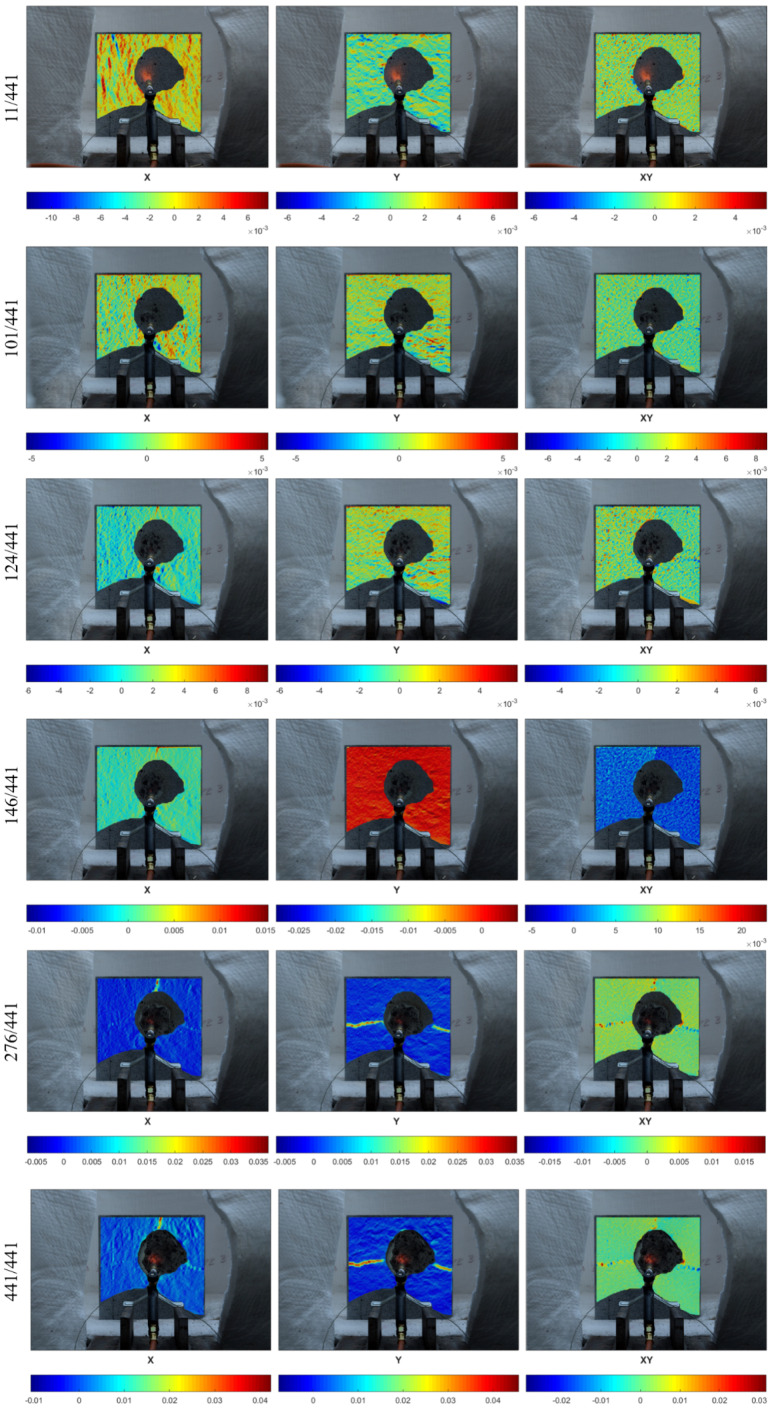
Deformation fields in the x- and y-directions for the largest specimen (300 × 300 mm).

Figure 8 presents the deformation fields in the x- and y-directions for the medium specimen (200 × 200 mm), while Figure 9 shows the results for the largest specimen (300 × 300 mm) during the small flame test. In the deformation fields, a red color means expansion while a blue one indicates contraction.

In the case of the medium specimen, 345 pictures were taken during the fire exposure period, while in the case of the large specimen, 441 pictures were collected. The six pictures presented for the medium (Figure 8) and large (Figure 9) specimens were chosen as representatives of different points in the period of fire exposure. Moreover, the presented points in time were very close to the time of the spalling event. In the case of the medium specimen, the pictures with the numbers 31/354 and 171/354 were taken at the exact time of the spalling event. Similarly, for the large specimen, the pictures with the numbers 11/441 and 146/441 represent the spalling event. In the pictures, it can be seen that during heating, the surface was divided into expanding and contracting lanes, in both the x- and y-directions. Such behavior of the heated side continued until the spalling event stopped. When it stopped, the deformations were regulated. Furthermore, the previously created cracks in the surrounding unexposed part of concrete continued to develop and the remaining area worked in compression.

**Figure 10:**
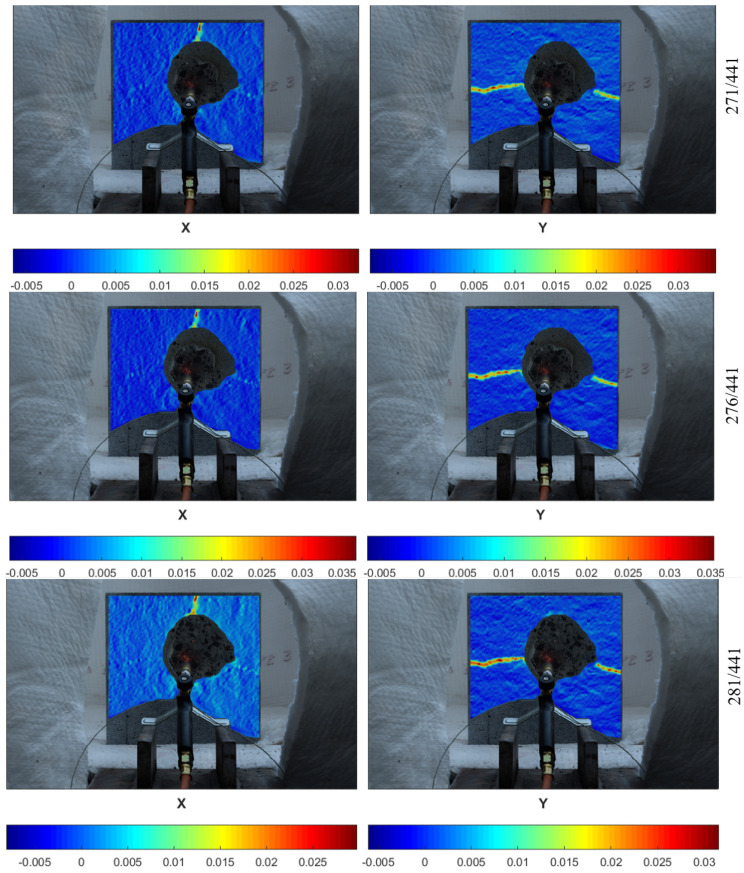
Deformation fields in the x- and y-directions for the medium specimen (200 × 200 mm).

**Figure 11:**
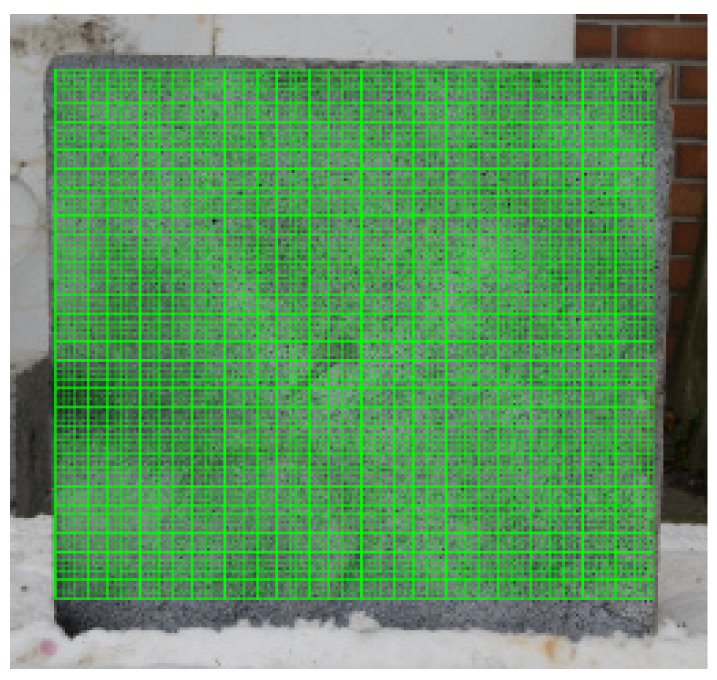
Grid of subsets in the unheated surface.

Moreover, the deformation fields of the exact spalling event show that the very early spalling event in the form of aggregate popcorn spalling provided slightly enhanced deformations in at least one direction (Figure 8—31/354 and Figure 9—11/441). However, when it comes to surface spalling (Figure 7—171/345, and Figure 8—146/441), it was characterized by a greater intensity and continuous form and took place at a later stage of the fire exposure, with the deformation field colors in red (tension) being displayed across the entire surface. Such deformation fields are unique within all 345 pictures for the smaller specimen and 441 pictures for the larger specimen. It should be emphasized that in the case of the smaller specimen, the picture 171/345 was taken exactly during the spalling event, while for the larger specimen, the picture 146/441 was taken just after it. This may explain why the deformation field in the x-direction for the larger specimen is not colored in red—it has already returned to the natural stage of deformation.

Finally, explosive spalling took place in the larger specimen just after the picture 276/441. It can be seen that the deformation observed in the neighboring pictures (271/441 and 281/441) was stable, which confirms that explosive spalling resulted from internal water pore pressure rather than thermal stress (Figure 10).

Due to the severe conditions of the test as caused by the high temperature and the almost unpredictable behavior of the concrete surface that was exposed to fire, the use of standard strain sensors was impossible at the heated side. Moreover, tracking the deformation of almost the entire surface was not possible using any contact method of measurement. Hence, the DIC method was effective and produced new results in our research on the spalling of concrete due to fire exposure.

### 3.2. Deformation of the Unheated Side

The unheated side of a specimen is the most frequently analyzed element during a spalling test. This is mostly caused by the configurations of experimental setups that let only the unheated part of an investigated specimen be observed [1]. In the case of the presented research, the unheated side was analyzed in the entire area. Figure 11 presents the grid subset within which the deformation fields were calculated. The authors did not experience any obstacles to successfully taking pictures within the fire exposure time.

**Figure 12:**
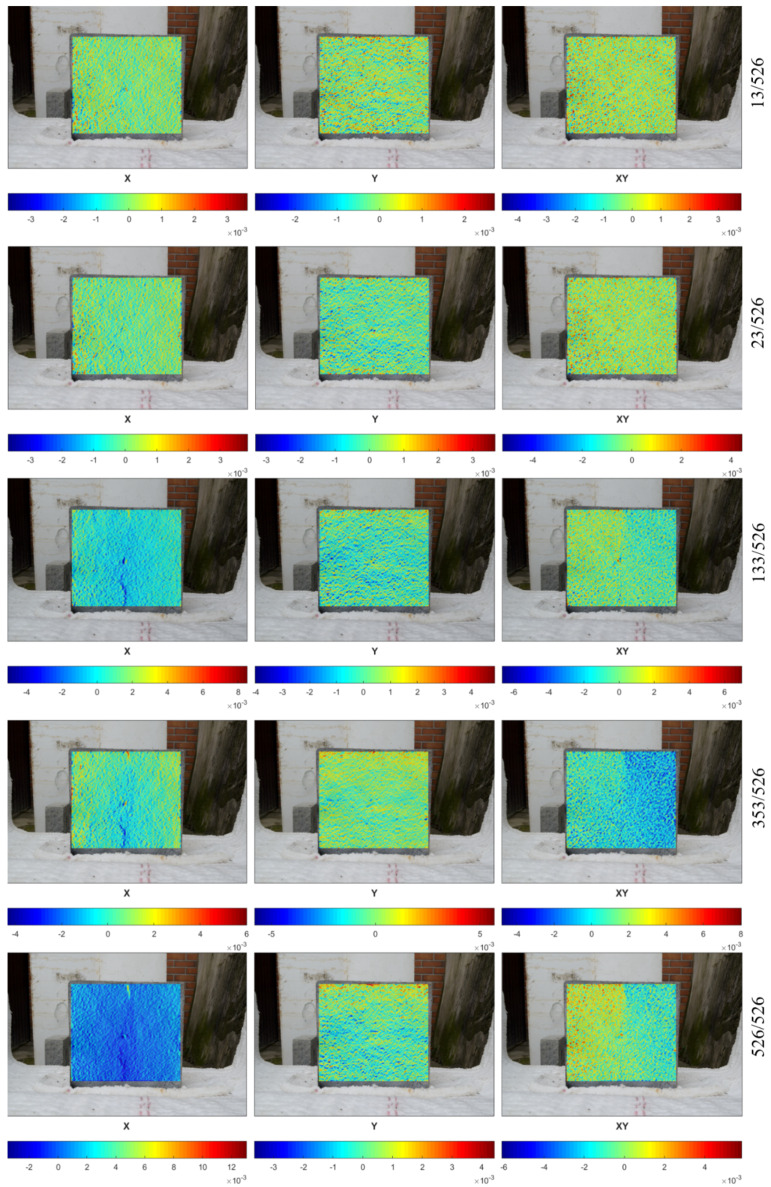
Deformation fields in the x- and y-directions in the unheated surface of specimen 1.

Figure 12 presents the deformation fields obtained for the opposite side of the tested specimen. During fire exposure, the deformation of the back surface developed in a similar manner to that of the fire exposed side. Until the time of the occurrence of the first crack, the surface was divided into expanding and contracting lanes. Moreover, in the x-direction, when a microcrack was formed at the top of the specimen, a compression zone appeared in the bottom part of the specimen. This observation was made for 2 of 2 tested specimens. Therefore, it is claimed that the cause of such deformation was the test configuration. The specimen lay on the ceramic wool that insulated the specimen from water evaporation. As a result, evaporated water moved toward the upper part of the specimen, enhancing the crack creation on the top of the slab. This could also be confirmed by the deformation fields in the y-direction, where the expanding and contracting lanes were concentrated in the upper part of the element.

The CivEng DIC method was successfully employed to measure the deformation of concrete exposed to fire at the unexposed side. The method allowed not only the deformation field across the entire surface and at any time of the test to be measured but also provided a reliable method to measure the development of cracking, including micro cracks.

### 3.3. Crack Width

Generally, in standard tests, the development of cracks and their width is mostly measured by an extensometer or LVDT. However, the crack location while testing is hard to predict and the crack pattern is impossible to assume before the test. The location of cracks differs from one test to another. An experienced researcher can predict the possible pattern of cracks, but the exact location is impossible to point out before the cracks start to develop. What is more, visual inspection is only possible when the crack starts to be visible with the naked eye, which occurs long after the creation of a crack. Finally, with the use of a contact method, it is impossible to capture the exact length, width, and number of cracks as the points to be analyzed are limited. Therefore, all of the contact methods for measuring crack width may be burdened with a high level of inaccuracy.

What is more, the standard method sometimes relies on the measurement of cracks as the tension of other devices, so any slip of gauges glued to the concrete surface is counted as the crack width. In the DIC method, the concrete surface itself is measured, so the accuracy of this method is only limited by the resolution of the digital camera.

**Figure 13:**
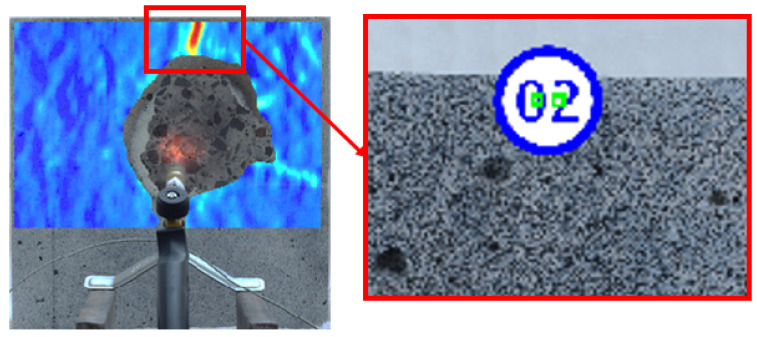
Virtual extensometer used for crack width measurement.

With the use of the DIC method, the cracking process is estimated during the post-processing stage. As a result, the exact number, pattern, and development of cracks within the entire test can be presented. Moreover, with the use of a virtual extensometer, the evolution of the crack width can be measured. This is obtained by measuring the changing distance between two points that are near enough to both sides of the crack development (Figure 13). The conversion from pixels to metric units is done by calculating the proportion between the length of the known object that is specified in standard length units (e.g., ruler) and its length in the image in pixels.

**Figure 14:**
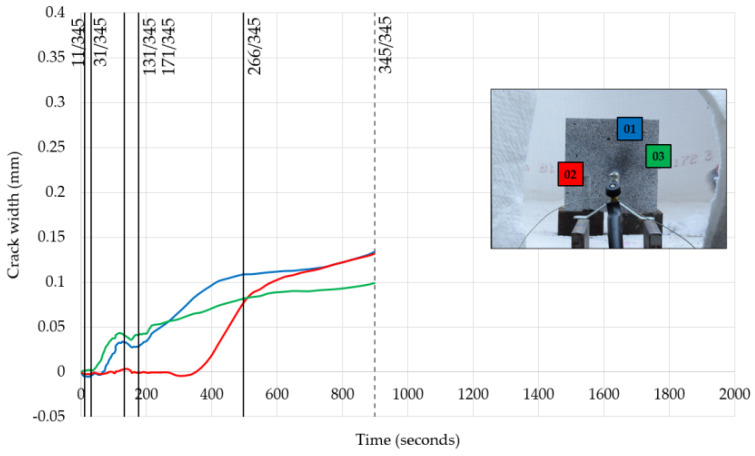
Evolution of crack width for the specimen with a size of 200 × 200 mm.

**Figure 15:**
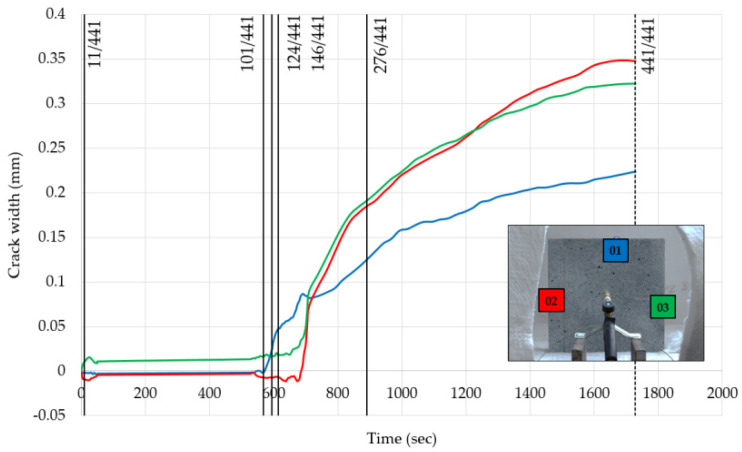
Evolution of crack width for the specimen with a size of 300 × 300 mm.

**Figure 16:**
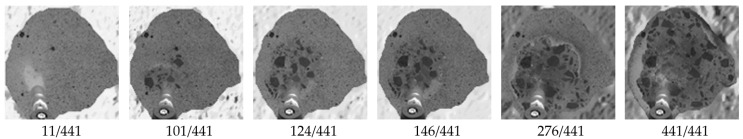
Spalling pattern for six points during fire exposure for the specimen with a size of 200 × 200 mm.

**Figure 17:**
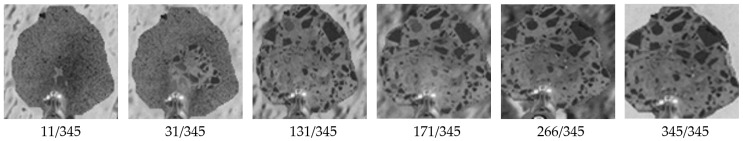
Spalling pattern for six points during fire exposure for the specimen with a size of 300 × 300 mm.

In Figure 14 and Figure 15, the evolution of crack width for the smaller and larger specimens is presented, respectively, while in Figure 16 and Figure 17, the spalling pattern for six points during the period of fire exposure is shown. Those six points in time are also indicated in diagrams of the cracking development.

Three cracks for both specimens were seen to have developed during the tests. Therefore, for each specimen, three virtual extensometers were employed to measure their evolution.

As can be seen in the diagrams, at least one crack started to develop in the first few minutes of fire exposure. It can be seen that the sensitivity of the measurement method was very high. Not only the process of crack creation but also the end of this process were made visible by the virtual extensometer. It can also be observed that the development of cracks increased when the more frequent surface spalling events stopped. Finally, as the diagram for the larger specimen shows, explosive spalling (no. 276/441) did not affect crack development.

### 3.4. Total Expansion of the Specimen

**Figure 18:**
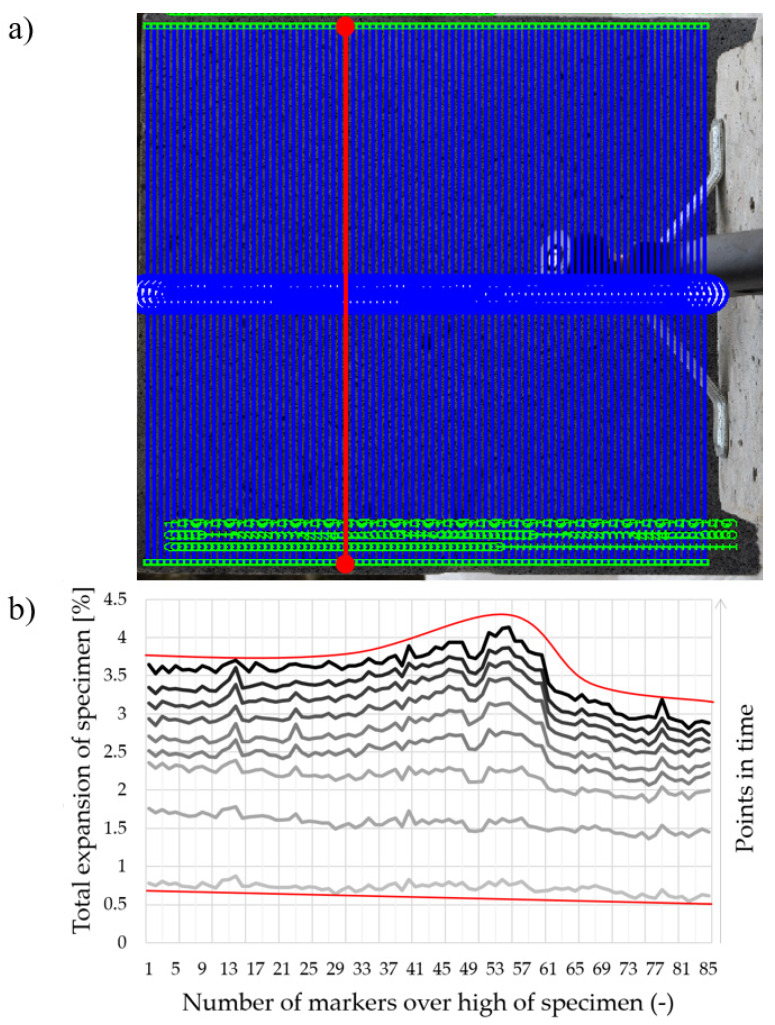
Measurement of the total expansion of the specimen: (**a**) two markers linked together to measure the distance between them, and (**b**) results of the analysis of the total expansion of the specimen along its height and over time.

Finally, the DIC method also allows any two points of the tested specimen to be linked and the change in the distance between them to be analyzed. The authors proposed the calculation for the total expansion of the specimen along its height during the test. For this purpose, two markers were linked together: one from the left edge and one from the right edge (the scheme of this procedure is shown in Figure 18a as two red connected dots). The subsequent linked markers had a distance of ca. 3.5 mm. As a result, the 85 pairs of markers were analyzed (in blue in Figure 18a) Furthermore, several points in time during the fire exposure were recorded.

Figure 18b presents the results of an analysis of the total expansion of the specimen along its height and over time. It can be seen that within the first few minutes of the test, the specimen expanded uniformly; however, the top side expanded a little more than the bottom part of the specimen. Furthermore, when surface spalling took place, the central part of the specimen started to expand significantly. Finally, the central part was more expanded than the surrounding part of the concrete. As in the later stage of the test, the crack appeared in the top part of the specimen, and it could be naturally observed that the top side was more expanded than the bottom part.

Such measurements may be helpful during an analysis of the uniformity of the distributed fire activity across the surface of a specimen or they may confirm if the boundary conditions were kept the same across the sample. In the presented case, the bottom part of the concrete was insulated with ceramic wool and was also the site that supported the specimen. As a result, the total expansion of the specimen could not be symmetrical along the specimen height.

## 4. Conclusions

The DIC method is a reliable method for analyzing the deformation of fire-exposed concrete. The authors did not encounter any obstacles when using the CivEng DIC method successfully during the tests—neither evaporated water nor heated air hindered the production of sharp pictures.

It was experimentally proven that it is possible to analyze the deformation of both the unheated and fire-exposed sides of concrete. What is more, the major advantage of the DIC method is that it can be used to analyze crack patterns as well as their development. This is a definite advantage that distinguishes the DIC method from other known contact methods.

Such measurement may be employed in further tests on a larger scale to analyze the behavior of concrete under fire exposure.

## Figures and Tables

**Table 1:** Compositions of the tested concrete.

Component	Unit	Amount
Cement CEM III/A 42.5 N Małogoszcz	kg/m^3^	482
Water	dm^3^/m^3^	145
w/c ratio	-	0.30
Dwudniaki river sand 0–2 mm	kg/m^3^	662
Gracze basalt agg. 2–8 mm	kg/m^3^	709
Gracze basalt agg. 8–16 mm	kg/m^3^	648
Plasticizer BASF BV 18	% mc	0.90
Superplasticizer BASF SKY 591	% mc	2.35
Cement paste	dm^3^/m^3^	300
Mortar	dm^3^/m^3^	550

**Table 2:** Initial properties of tested concrete (mean values).

Property	Unit	Basalt
Apparent density	kg/m^3^	2533.2
Water content	%	2.7
Permeability (k_c_)	m^2^	5.23 × 10^−18^
Compressive strength	MPa	96.2
Modulus of elasticity	GPa	48.9
